# Gastrointestinal Kaposi Sarcoma as the Initial Presentation of Undiagnosed HIV Infection: A Case of Unexplained Anemia

**DOI:** 10.1155/crgm/1526999

**Published:** 2026-11-03

**Authors:** Amir Omar, Tracy Zouein, Rayane Salameh, Karim Zodeh, Karam Karam, Georges Aftimos, Pierre Hani

**Affiliations:** ^1^ Department of Gastroenterology University of Balamand Beirut Lebanon balamand.edu.lb; ^2^ Department of Internal Medicine University of Balamand Beirut Lebanon balamand.edu.lb; ^3^ Department of Infectious Disease Saint George University Hospital Medical Center Beirut Lebanon; ^4^ Department of Pathology University of Balamand Beirut Lebanon balamand.edu.lb

**Keywords:** gastrointestinal involvement, human herpesvirus-8 (HHV-8), human immunodeficiency virus (HIV), Kaposi sarcoma, unexplained anemia

## Abstract

Kaposi sarcoma (KS) is an angioproliferative malignancy associated with human herpesvirus‐8 (HHV‐8) infection and is strongly linked to immunosuppression, particularly in the setting of human immunodeficiency virus (HIV). Although cutaneous involvement is the most common presentation, visceral disease—especially within the gastrointestinal (GI) tract—may occur and is frequently underrecognized due to its often asymptomatic or nonspecific clinical manifestations. We report the case of a 41‐year‐old male who presented with severe, unexplained anemia without overt GI bleeding. Endoscopic evaluation revealed multiple violaceous, polypoid lesions throughout the stomach, duodenum, and colon. Histopathological examination, supported by immunohistochemical staining positive for CD31, CD34, and HHV‐8, confirmed the diagnosis of KS. Subsequent HIV testing was strongly reactive, with confirmatory HIV‐1 RNA testing subsequently requested. This case highlights the diagnostic challenge posed by GI KS and underscores the importance of maintaining a high index of suspicion in patients presenting with unexplained anemia. Early recognition through comprehensive endoscopic and histopathological evaluation is critical, as it may reveal previously undiagnosed HIV infection and significantly influence clinical management and prognosis.

## 1. Introduction

Kaposi sarcoma (KS) is an angioproliferative neoplasm caused by infection with human herpesvirus‐8 (HHV‐8) and is strongly associated with immunosuppression, particularly in individuals with human immunodeficiency virus (HIV) infection. Although the incidence of AIDS‐related KS has markedly declined following the widespread use of combination antiretroviral therapy (cART), it remains an AIDS‐defining malignancy and continues to occur in patients with undiagnosed, untreated, or advanced HIV infection, especially in regions where HHV‐8 prevalence is high [1–4].

The pathogenesis of KS involves HHV‐8 infection of endothelial cells, resulting in dysregulated angiogenesis, spindle‐cell proliferation, chronic inflammation, and resistance to apoptosis. However, HHV‐8 infection alone is insufficient for tumor development; impaired immune surveillance and persistent immune activation play essential roles in disease progression [4].

Although KS most commonly presents with cutaneous lesions, visceral involvement is well recognized, with the gastrointestinal (GI) tract representing the most frequent extracutaneous site [1, 2, 5]. GI KS may affect any segment of the digestive tract, particularly the stomach and small intestine. Endoscopic findings are variable and include flat, nodular, polypoid, or ulcerative violaceous lesions. Because GI involvement is frequently asymptomatic or manifests with nonspecific symptoms such as abdominal pain, weight loss, occult GI bleeding, or anemia, diagnosis is often delayed, making a high index of clinical suspicion essential [1, 5].

We report a case of GI KS presenting as severe anemia of unclear etiology at presentation, which ultimately led to the diagnosis of previously unrecognized HIV infection. This case highlights the importance of considering GI KS in the differential diagnosis of unexplained anemia and demonstrates the pivotal role of endoscopic evaluation and histopathological confirmation in establishing an early diagnosis.

## 2. Case Presentation

A 41‐year‐old Caucasian male presented to the emergency department with a two‐week history of progressive fatigue, dizziness, and decreased oral intake. He additionally reported a four‐day history of dry cough and rhinorrhea, without associated fever, chills, or known sick contacts. He denied nausea, vomiting, hematochezia, or melena.

The patient had no known past medical history. His surgical history was notable only for excision of a pilonidal cyst 10 years prior. He reported occasional tobacco use and social alcohol consumption without binge drinking. He denied the use of prescription medications, over‐the‐counter nonsteroidal anti‐inflammatory drugs (NSAIDs), supplements, hormones, or illicit substances.

The patient reported prior sexual activity with multiple partners, both protected and unprotected, and denied any known history of sexually transmitted infections. He had no known drug or food allergies.

On physical examination, he appeared thin, pale, and fatigued but in no acute distress. A single painless, nonblanching macular cutaneous lesion was observed on the posterior neck near the left shoulder. Cardiopulmonary examination revealed normal heart sounds without murmurs and clear lung fields. The abdomen was soft, nondistended, and nontender, with no evidence of lymphadenopathy or hepatosplenomegaly. The remainder of the examination was unremarkable.

Initial laboratory investigations obtained before blood transfusion demonstrated severe normocytic anemia, with a hemoglobin level of 6.3 g/dL, hematocrit of 21.5%, MCV of 92.7 fL, and RDW of 18.9%. The patient denied melena or hematochezia, and no overt GI bleeding was documented. The iron profile showed low serum iron, reduced total iron‐binding capacity, and markedly elevated ferritin, consistent with a substantial inflammatory component to the anemia.

Biochemical analysis demonstrated normal renal function and electrolytes. Total protein was 72 g/L, with hypoalbuminemia (26 g/L) and elevated globulins (46 g/L). This pattern, characterized by hypoalbuminemia with relative hyperglobulinemia, was considered compatible with the systemic inflammatory and immune activation context of the patient’s underlying disease. Liver function tests were within normal limits.

Evaluation of anemia revealed low serum iron (31 µg/dL), decreased total iron‐binding capacity (156 µg/dL), and markedly elevated ferritin (1577.6 ng/mL). Folate and vitamin B12 levels were within normal limits. Reticulocyte count was 2.2%, with normal lactate dehydrogenase and haptoglobin levels. Peripheral blood smear demonstrated anisocytosis and hypochromia, with an adequate platelet count. Direct and indirect Coombs tests were negative.

The patient was initiated on intravenous esomeprazole (40 mg twice daily), transfused two units of packed red blood cells, and admitted for further evaluation. Repeat laboratory testing on hospital Day 2 demonstrated improvement in hemoglobin to 9.4 g/dL, persistent lymphopenia, and an increase in CRP to 24 mg/L.

Esophagogastroduodenoscopy revealed no stigmata of recent bleeding but demonstrated multiple reddish‐purple polypoid lesions distributed throughout the gastric mucosa, including the cardia, fundus, incisura, body, and both curvatures, with lesser involvement of the antrum (Figure 1A–E). The lesions were friable and exhibited mild bleeding following biopsy.

**FIGURE 1:**
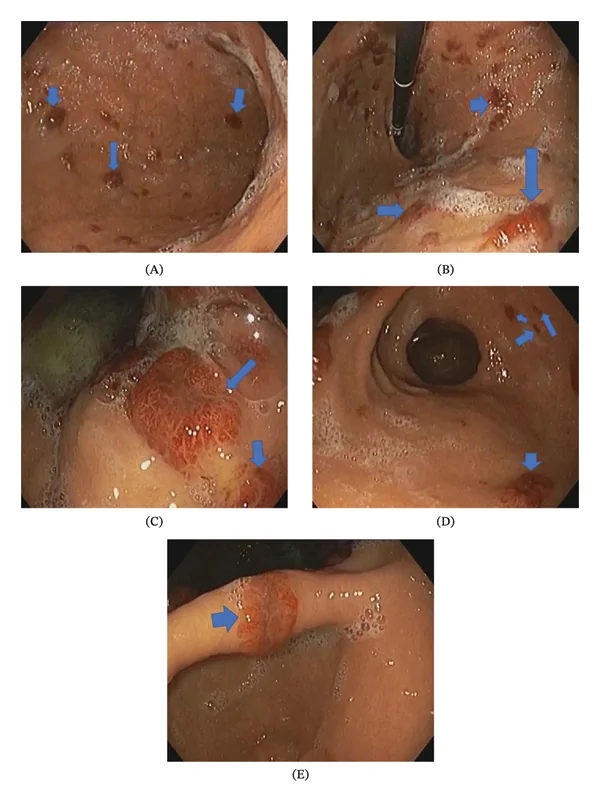
Gastric Kaposi sarcoma lesions. (A) Multiple reddish‐purple polypoid lesions involving the gastric body (blue arrows). (B) Multiple violaceous lesions involving the gastric body and fundus, visualized in retroflexion (blue arrows). (C) Multiple lesions involving the proximal gastric body and cardia (blue arrows). (D) Violaceous polypoid lesions involving the gastric antrum (blue arrows). (E) Violaceous lesion involving the angularis (blue arrow).

A similar lesion was identified in the second portion of the duodenum (Figure 2), and biopsies were obtained. Colonoscopy demonstrated comparable lesions in the rectum and sigmoid colon extending up to 20 cm from the anal verge (Figure 3), with the remainder of the colon and terminal ileum appearing normal.

**FIGURE 2:**
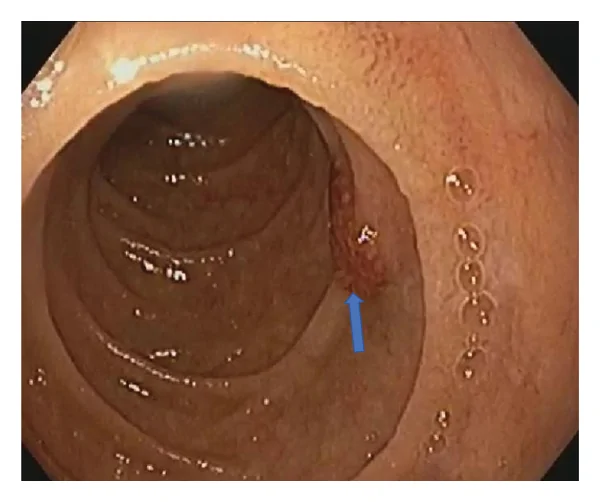
Duodenal Kaposi sarcoma lesion. Single reddish‐purple/violaceous lesion identified in the second portion of the duodenum (D2) (blue arrow).

**FIGURE 3:**
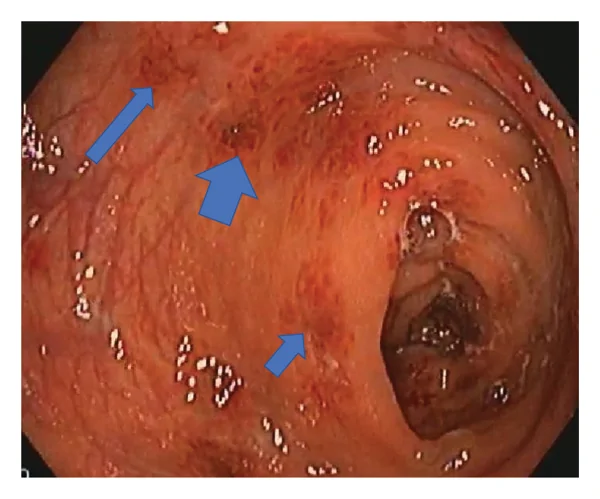
Rectosigmoid Kaposi sarcoma lesions. Multiple reddish‐purple/violaceous lesions involving the rectum and sigmoid colon, extending proximally to approximately 20 cm from the anal verge.

Computed tomography of the chest, abdomen, and pelvis revealed multiple nodular and ground‐glass opacities in the left lung base with tree‐in‐bud appearance, suggestive of atypical pneumonia (Figure 4), along with mediastinal and peripheral lymphadenopathy. Abdominal imaging demonstrated splenomegaly (16.5 cm), prominent portal and splenic veins, and multiple small retroperitoneal and mesenteric lymph nodes (Figure 5).

**FIGURE 4:**
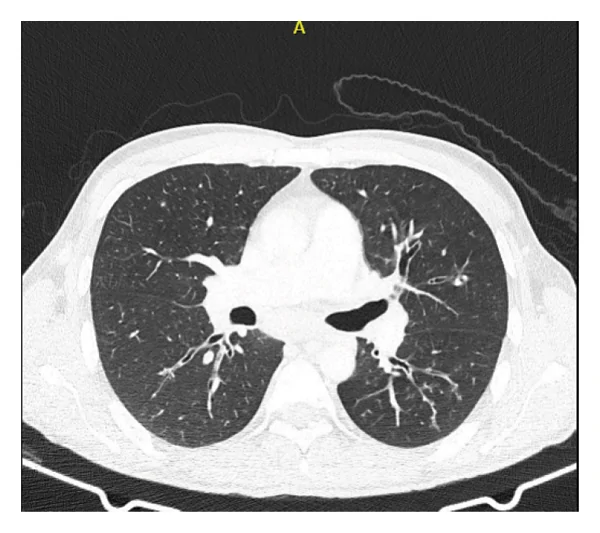
Chest computed tomography findings. Computed tomography of the chest demonstrating multiple nodular and ground‐glass pulmonary opacities with a tree‐in‐bud appearance at the left lung base, associated with mediastinal and peripheral lymphadenopathy. These findings were considered suspicious for an infectious or inflammatory pulmonary process; pulmonary Kaposi sarcoma was not histologically confirmed.

**FIGURE 5:**
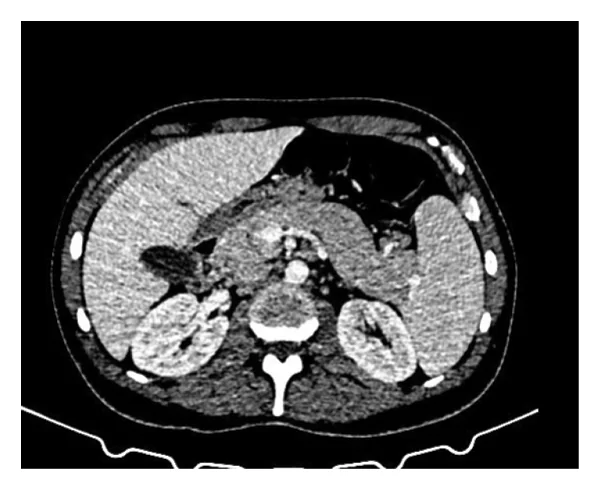
Abdominal computed tomography findings. Computed tomography demonstrating splenomegaly (16.5 cm), prominent portal and splenic veins, and multiple small retroperitoneal and mesenteric lymph nodes.

During hospitalization, the patient developed worsening cough and low‐grade fever. Bronchoscopy with bronchoalveolar lavage yielded growth of methicillin‐sensitive *Staphylococcus aureus*, for which he was treated with oral cephalexin (Figure 6).

**FIGURE 6:**
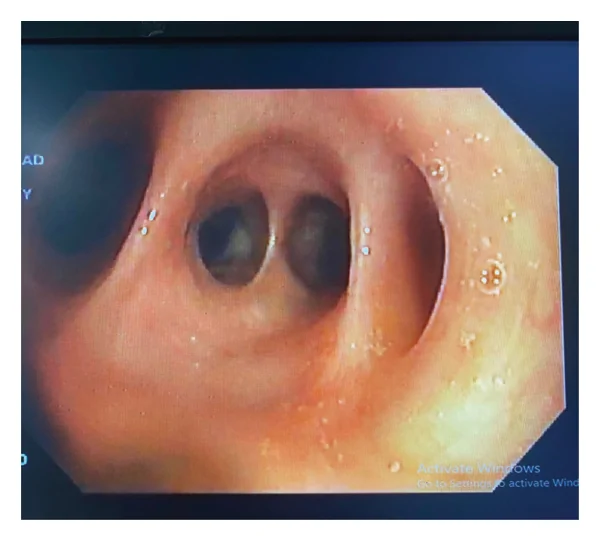
Bronchoscopy: Bronchoscopic examination without an endoscopically apparent endobronchial Kaposi sarcoma lesion. Bronchoalveolar lavage subsequently yielded methicillin‐sensitive *Staphylococcus aureus*.

Histopathological evaluation of GI biopsies revealed spindle‐cell proliferation within the lamina propria, associated with inflammatory infiltrates and slit‐like vascular spaces. Immunohistochemical staining demonstrated positivity for CD31, CD34, and HHV‐8, confirming the diagnosis of KS (Figures 7, 8, 9, 10, 11, 12, 13, 14, 15, 16, 17, and 18).

**FIGURE 7:**
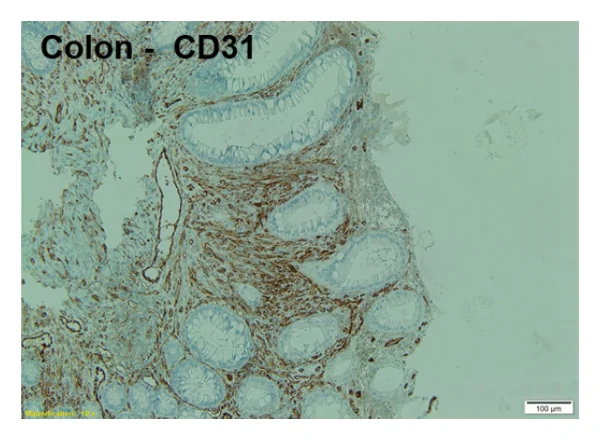
Immunohistochemical staining for CD31 demonstrating positive staining of the vascular endothelial component of the lesion, supporting its vascular differentiation.

**FIGURE 8:**
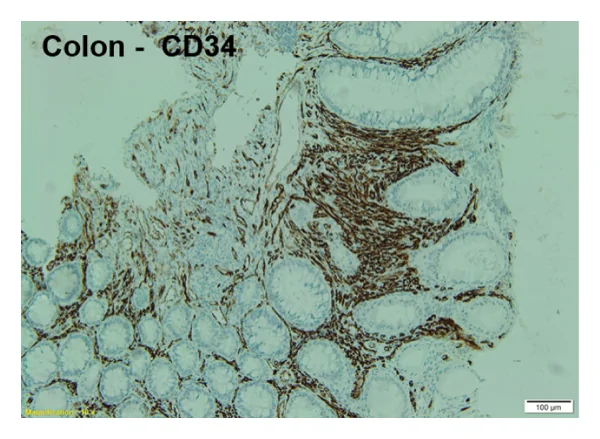
Immunohistochemical staining for CD34 showing positivity in the lesional spindle cells and vascular structures, supporting the diagnosis of Kaposi sarcoma.

**FIGURE 9:**
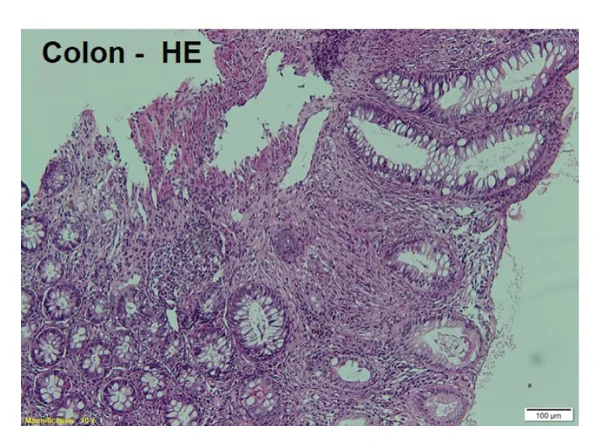
Higher‐power H&E image showing spindle‐cell proliferation, vascular channel formation, and associated inflammatory cell infiltrates, characteristic of Kaposi sarcoma.

**FIGURE 10:**
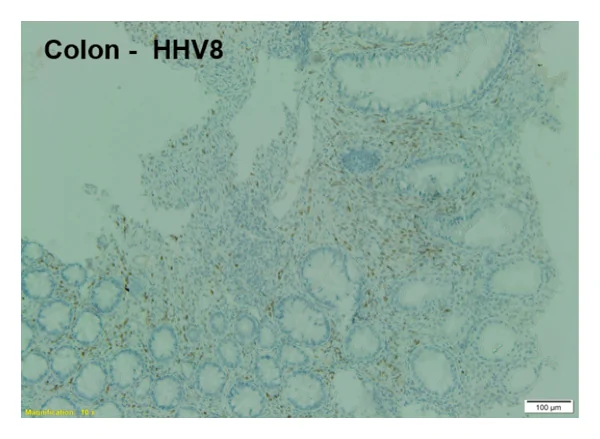
Immunohistochemical staining for HHV‐8 demonstrating nuclear positivity in lesional cells, providing specific support for the diagnosis of Kaposi sarcoma.

**FIGURE 11:**
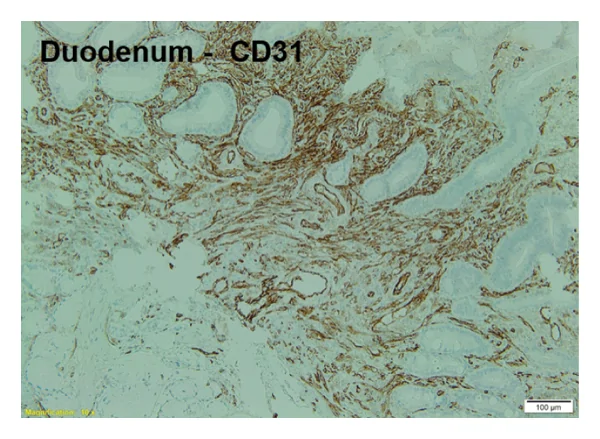
Immunohistochemical staining for CD31 demonstrating positive staining of the vascular endothelial component of the lesion, supporting its vascular differentiation.

**FIGURE 12:**
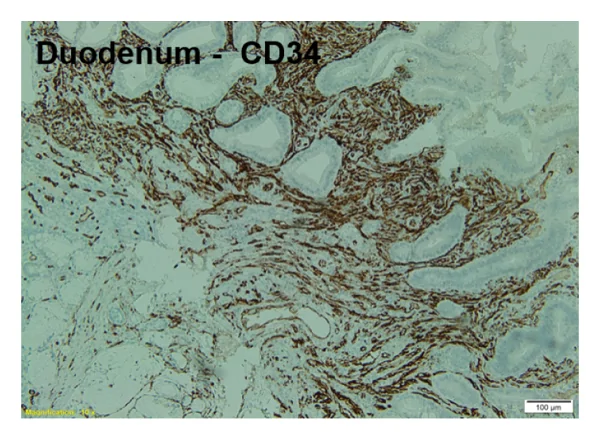
Immunohistochemical staining for CD34 showing positivity in the lesional spindle cells and vascular structures, supporting the diagnosis of Kaposi sarcoma.

**FIGURE 13:**
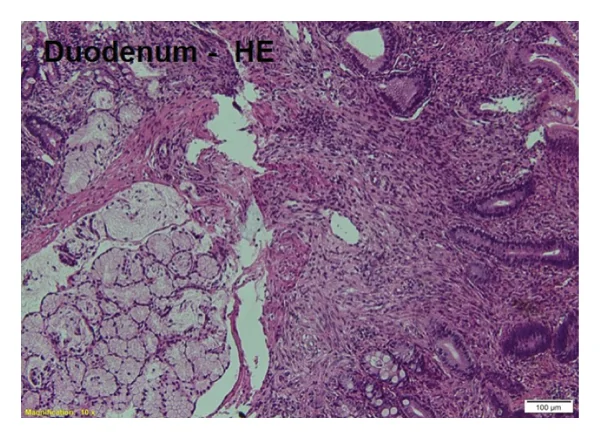
H&E staining of a gastrointestinal biopsy demonstrating spindle‐cell proliferation and irregular vascular spaces within the lamina propria.

**FIGURE 14:**
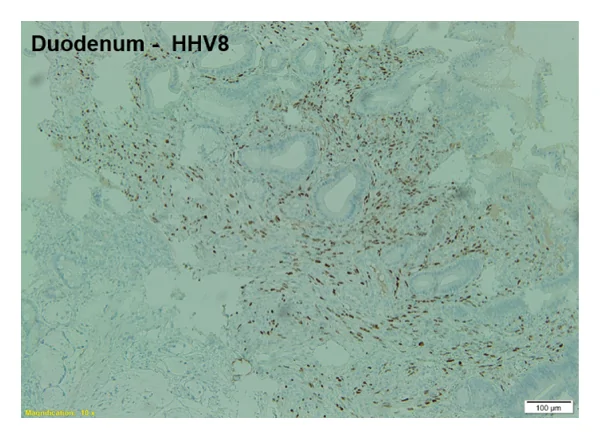
Higher‐power histological examination highlighting the characteristic slit‐like vascular spaces and spindle‐cell morphology of Kaposi sarcoma.

**FIGURE 15:**
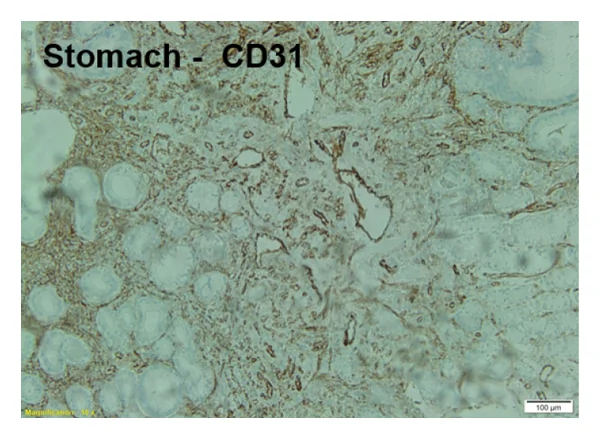
Immunohistochemical staining for CD31 showing endothelial marker expression within the lesional vascular structures.

**FIGURE 16:**
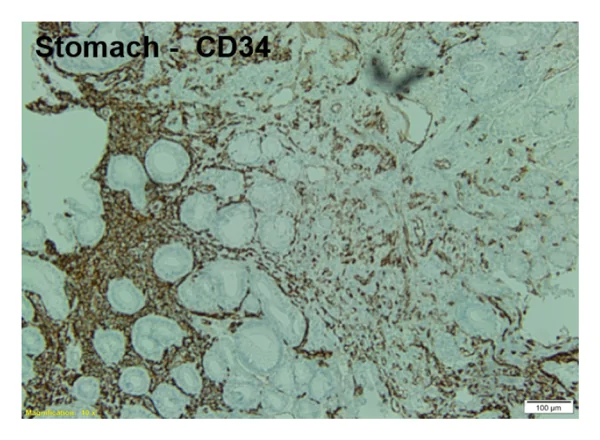
Immunohistochemical staining for CD34 demonstrating positive staining of the vascular and spindle‐cell components of the lesion.

**FIGURE 17:**
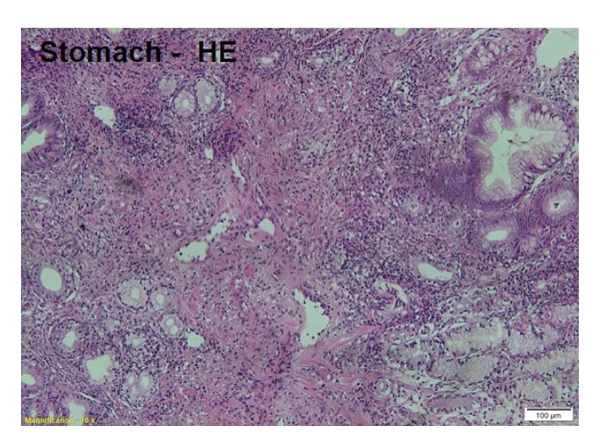
H&E staining of a gastrointestinal biopsy demonstrating spindle‐cell proliferation and irregular vascular spaces within the lamina propria.

**FIGURE 18:**
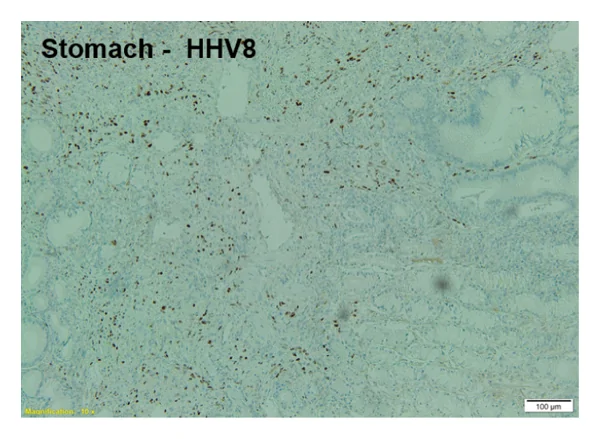
Representative histopathological and immunohistochemical findings demonstrating the characteristic morphology and immunophenotype of gastrointestinal Kaposi sarcoma.

Biopsies obtained from the gastric, duodenal, and rectosigmoid lesions demonstrated similar histopathological features, with spindle‐cell proliferation within the lamina propria, inflammatory infiltrates, and slit‐like vascular spaces. Immunohistochemical staining was positive for CD31, CD34, and HHV‐8, confirming KS in the sampled GI sites.

Subsequent HIV‐1/2 serologic testing returned a strongly reactive result (495.4; reference > 1.00), and confirmatory polymerase chain reaction testing was requested. Confirmatory HIV‐1 RNA testing was requested following the strongly reactive HIV‐1/2 serologic result; however, the confirmatory result was not available in the records accessible to the authors. Similarly, CD4+ T‐cell count, CD4 percentage, and HIV viral load were not available. Hepatitis B and C serologies, subsequent HIV staging, and additional opportunistic infection testing were also unavailable. The patient was referred to the Infectious Diseases service for further evaluation and management, including the initiation of antiretroviral therapy; however, subsequent treatment details and clinical follow‐up were not available to the authors.

The diagnosis of KS, an AIDS‐defining illness, prompted urgent referral to the Infectious Diseases service for further immunologic evaluation, including CD4+ T‐cell count and HIV viral load, and the initiation of cART.

Although GI KS lesions were friable and demonstrated mild bleeding following biopsy, the available data did not establish chronic occult GI blood loss as the cause of the severe anemia. The anemia was, therefore, considered severe and likely multifactorial in the context of systemic inflammation and underlying HIV‐associated disease. The patient subsequently received two units of packed red blood cells, with hemoglobin increasing to 9.4 g/dL on hospital Day 2.

Although the patient had a solitary violaceous cutaneous lesion and imaging demonstrated mediastinal lymphadenopathy and pulmonary nodular opacities, biopsies of these sites were not performed. Therefore, while these findings were considered suspicious for extracutaneous KS in the clinical context, histopathological confirmation was obtained only from the GI lesions.

Despite the absence of melena or hematochezia, the severity of the symptomatic anemia, with a hemoglobin level of 6.3 g/dL, prompted further investigation for an underlying cause. The iron profile demonstrated low serum iron, reduced total iron‐binding capacity, and markedly elevated ferritin in the setting of an inflammatory response, suggesting a substantial inflammatory component to the anemia. No alternative obvious source of the profound anemia was identified from the initial clinical evaluation. Given the severity of the anemia and the possibility of occult GI pathology, inpatient endoscopic evaluation was pursued. Esophagogastroduodenoscopy subsequently revealed multiple reddish‐purple, polypoid and friable lesions throughout the stomach and a similar lesion in the second portion of the duodenum. Colonoscopy demonstrated comparable lesions involving the rectum and sigmoid colon. Biopsies ultimately established the diagnosis of GI KS.

The patient presented with severe symptomatic normocytic anemia. Although GI KS lesions were friable and demonstrated mild bleeding following biopsy, the available clinical data did not establish chronic GI blood loss as the cause of the anemia. The anemia was, therefore, considered severe and likely multifactorial, with a substantial inflammatory component.

Although a solitary violaceous cutaneous lesion was identified and chest imaging demonstrated pulmonary nodular and ground‐glass opacities with associated lymphadenopathy, neither the cutaneous nor pulmonary findings was biopsied. Therefore, these findings remain suspicious for extracutaneous KS but were not histologically confirmed. Histopathological confirmation of KS was obtained exclusively from the GI lesions.

Following the diagnosis of GI KS and newly identified HIV infection, the patient was referred to the Infectious Diseases service for further evaluation and management. However, subsequent follow‐up information was not available to the authors. Therefore, the initiation and specific regimen of antiretroviral therapy, the use of systemic chemotherapy, subsequent CD4+ T‐cell count and HIV viral load, serial hemoglobin measurements, and repeat endoscopic evaluation were unavailable. The last documented hemoglobin level was 9.4 g/dL following transfusion of two units of packed red blood cells. Consequently, the subsequent clinical response, evolution of the GI lesions, duration of follow‐up, and final patient outcome could not be determined.

## 3. Discussion

KS is an angioproliferative neoplasm driven by infection with HHV‐8, with its development strongly associated with immunosuppression, particularly in individuals with HIV [144]. Although four clinical variants have been described—classic, endemic, iatrogenic, and epidemic—the latter remains the most aggressive form and is closely linked to advanced HIV infection [5, 6]. Despite a marked decline in incidence following the introduction of cART, KS continues to be encountered, particularly in patients with undiagnosed or untreated HIV infection, and remains an AIDS‐defining illness [7, 8].

The pathogenesis of KS reflects a complex interaction between viral oncogenesis and host immune dysfunction. HHV‐8 infection of endothelial cells promotes tumorigenesis through activation of angiogenic pathways, inhibition of apoptosis, and induction of spindle‐cell proliferation [9]. Viral proteins, including latency‐associated nuclear antigen, facilitate immune evasion and persistence of infection, while proangiogenic factors such as vascular endothelial growth factor further contribute to lesion development [9]. Nevertheless, HHV‐8 infection alone is insufficient for tumor formation, and additional factors—particularly immune suppression and chronic inflammation—are required [10].

GI involvement represents the most common form of visceral KS, occurring in up to 50% of the patients with AIDS‐related disease [1, 2]. The stomach and small intestine are most frequently affected although lesions may arise anywhere along the GI tract [2, 5]. Importantly, GI KS often remains clinically silent and may occur without cutaneous manifestations, making diagnosis particularly challenging [3, 5]. When present, symptoms are typically nonspecific and include abdominal pain, nausea, weight loss, or anemia, while overt bleeding or obstructive complications are less common [1, 2, 5].

Endoscopically, GI KS demonstrates considerable variability, ranging from flat or maculopapular lesions to nodular, polypoid, or ulcerated masses, often with a characteristic violaceous or reddish discoloration reflecting their vascular nature [5, 6]. These findings are not specific and may mimic a variety of neoplastic and infectious conditions, including GI stromal tumors, lymphoma, angiosarcoma, melanoma, or bacillary angiomatosis [6], thereby increasing the risk of misdiagnosis.

Histopathological examination remains the cornerstone of diagnosis [7]. Typical features include spindle‐cell proliferation with slit‐like vascular spaces and extravasated erythrocytes [6, 7]. However, diagnosis may be difficult in early or subtle lesions, and false‐negative results can occur, particularly with superficial biopsies, as lesions are frequently submucosal [8]. For this reason, obtaining multiple and adequately deep biopsy samples is essential when clinical suspicion persists. Immunohistochemistry plays a decisive role, with HHV‐8 latent nuclear antigen serving as a highly sensitive and specific marker, supported by endothelial markers such as CD31 and CD34 [7].

Our case highlights several important clinical considerations. The patient presented with severe, unexplained anemia in the absence of overt GI bleeding, indicating that occult blood loss may be the only manifestation of GI involvement [5]. Furthermore, the diagnosis of KS ultimately led to the identification of previously unrecognized HIV infection, emphasizing the need to consider underlying immunodeficiency in patients with atypical or unexplained GI findings. Although a subtle cutaneous lesion was present, the disease was predominantly visceral, underscoring that minimal skin involvement does not exclude extensive internal disease [5, 6].

The staging of AIDS‐related KS is commonly based on the AIDS Clinical Trials Group (ACTG) classification, which incorporates tumor burden, immune status, and systemic illness [11]. In the modern cART era, tumor extent and systemic involvement are the principal determinants of prognosis. Visceral disease, particularly involving the GI tract, is associated with more advanced disease and poorer outcomes if not promptly treated [3].

Management is guided by disease severity and immune status. Initiation or optimization of cART remains the cornerstone of therapy and can lead to significant regression of lesions through immune restoration [4, 10]. However, patients with extensive visceral involvement or significant symptoms often require systemic chemotherapy, even in the setting of virologically controlled HIV infection [12, 13]. Liposomal doxorubicin is considered first‐line treatment due to its efficacy and favorable safety profile, while paclitaxel represents an effective alternative in advanced or refractory cases [14, 15]. Clinicians should also be aware of immune reconstitution inflammatory syndrome, which may transiently worsen KS following the initiation of cART and should not be mistaken for disease progression [10].

Although the ACTG T/I/S classification is commonly used to assess the extent and severity of AIDS‐related KS, complete staging could not be performed in our patient because CD4+ T‐cell count and complete assessment of extra intestinal disease were unavailable. Furthermore, the pulmonary and cutaneous abnormalities were not biopsied and, therefore, remained suspected rather than confirmed sites of KS. Accordingly, no definitive ACTG T/I/S classification was assigned.

Although GI KS may be associated with GI bleeding and anemia, the mechanism underlying the severe anemia in our patient could not be established with certainty. In the absence of overt GI bleeding or objective evidence of chronic occult blood loss, the anemia should be considered associated with the overall disease context rather than definitively attributed to GI blood loss from KS. Therefore, the present case should be interpreted as severe anemia occurring in association with GI KS rather than definitive evidence of anemia caused by occult bleeding from KS.

A limitation of this report is the absence of complete longitudinal HIV‐related laboratory and clinical data, including CD4+ T‐cell count, HIV viral load, confirmatory HIV‐1 RNA results, and subsequent treatment and follow‐up information. Consequently, the degree of immunosuppression and virologic status at the time of KS diagnosis could not be quantitatively characterized. Another limitation of this case is the lack of longitudinal follow‐up after the diagnosis of GI KS and HIV infection. The absence of subsequent clinical, hematologic, immunologic, virologic, and endoscopic data prevented the assessment of treatment response, evolution of the GI lesions, and long‐term outcome.

This case illustrates that clinically significant GI KS may be present despite the absence of overt GI bleeding. In a patient presenting with severe unexplained anemia, the absence of melena or hematochezia should not preclude investigation for occult GI pathology. In our patient, the severity of the anemia prompted endoscopic evaluation despite the lack of overt bleeding, leading to the unexpected identification of characteristic GI lesions and ultimately to the diagnosis of KS and previously unrecognized HIV infection.

In summary, this case underscores the importance of considering GI KS in the differential diagnosis of unexplained anemia, even in the absence of known HIV infection or significant cutaneous disease. Early recognition through comprehensive endoscopic evaluation, adequate biopsy sampling, and appropriate immunohistochemical analysis is essential. Timely diagnosis not only enables appropriate oncologic management but may also reveal previously unrecognized, life‐threatening yet treatable underlying immunodeficiency, significantly altering patient outcomes.

## 4. Conclusion

This case emphasizes that GI KS may present with nonspecific manifestations such as unexplained anemia and minimal cutaneous involvement, thereby complicating timely diagnosis. Comprehensive endoscopic evaluation, adequate biopsy sampling, and confirmatory immunohistochemical analysis are essential for accurate identification. Recognition of visceral KS should prompt evaluation for underlying immunodeficiency, as it may reveal previously undiagnosed HIV infection. Early diagnosis facilitates timely initiation of combination antiretroviral therapy and, when indicated, systemic chemotherapy, both of which are critical for improving clinical outcomes and reducing morbidity and mortality.

## Author Contributions

Amir Omar: conceptualization, data curation, and writing the original draft.

Tracy Zouein: conceptualization, data curation, and writing the original draft.

Rayane Salameh: conceptualization, data curation, and writing the original draft.

Karim Zodeh: conceptualization, data curation, and writing the original draft.

Karam Karam: conceptualization, data curation, and writing the original draft.

Georges Aftimos: conceptualization, data curation, and writing the original draft.

Pierre Hani: conceptualization, data curation, and writing the original draft.

## Funding

No funding was received for this manuscript.

## Consent

A written informed consent was obtained from the patient for this case report and any accompanying images.

## Conflicts of Interest

The authors declare no conflicts of interest.

## Data Availability

Data used to support the findings of this study are available on request from the corresponding author.

## References

[1] Rammohan R. , Natt D. , Magam S. et al., S3759 Beyond the Skin: Unveiling Advanced Kaposi Sarcoma With Gastrointestinal Involvement in an HIV/AIDS Patient, American Journal of Gastroenterology. (2025) 120, no. 10S2, 10.14309/01.ajg.0001142496.91536.68.

[2] Suster D. I. , Rastegar S. , Salviato T. et al., Polypoid Kaposi Sarcoma Involving the Lower Gastrointestinal Tract: Clinicopathologic Study of 15 Cases, Archives of Pathology & Laboratory Medicine. (2025) 149, no. 6, 519–526, 10.5858/arpa.2024-0196-OA. 39246073

[3] Salehiazar S. , K M. B. , Salahi W. , and Qing X. , HIV Associated Kaposi’s Sarcoma With Visceral Involvement and Rectal Bleeding, American Journal of Clinical Pathology. (2023) no. 1, 10.1093/ajcp/aqad150.065.

[4] Grabar S. and Costagliola D. , Epidemiology of Kaposi’s Sarcoma, Cancers. (2021) 13, no. 22, 10.3390/cancers13225692. PMC8616388 34830846

[5] Nagata N. , Shimbo T. , Yazaki H. et al., Predictive Clinical Factors in the Diagnosis of Gastrointestinal Kaposi’s Sarcoma and Its Endoscopic Severity, PLoS One. (2012) 7, no. 11, 10.1371/journal.pone.0046967. PMC3511500 23226197

[6] Shore B. A. and Karamchandani D. M. , Nonclassic Histologic Variants of Kaposi Sarcoma With Nonspecific Endoscopic Findings in the Gastrointestinal Tract, Archives of Pathology and Laboratory Medicine. (2025) 149, no. 12, 1084–1092. 10.5858/arpa.2025-0085-OA 40522889

[7] Nagata N. , Igari T. , Shimbo T. et al., Diagnostic Value of Endothelial Markers and HHV-8 Staining in Gastrointestinal Kaposi Sarcoma and Its Difference in Endoscopic Tumor Staging, World Journal of Gastroenterology. (2013) 19, no. 23, 3608–3614, 10.3748/wjg.v19.i23.3608. PMC3691034 23801862

[8] Nagata N. , Sekine K. , Igari T. et al., False-Negative Results of Endoscopic Biopsy in the Diagnosis of Gastrointestinal Kaposi’s Sarcoma in HIV-Infected Patients, Pathology Research International. (2012) 2012, 854146–6, 10.1155/2012/854146. PMC3513736 23227427

[9] Bishop B. N. and Lynch D. T. , Kaposi Sarcoma, Statpearls [Internet], 2026, StatPearls Publishing, Treasure Island (FL), https://www.ncbi.nlm.nih.gov/books/NBK534839/.

[10] Schneider J. W. and Dittmer D. P. , Diagnosis and Treatment of Kaposi Sarcoma, American Journal of Clinical Dermatology. (2017) 18, no. 4, 529–539, 10.1007/s40257-017-0270-4. PMC5509489 28324233

[11] Dupin N. , Jary A. , Boussouar S. et al., Current and Future Tools for Diagnosis of Kaposi’s Sarcoma, Cancers. (2021) 13, no. 23, 10.3390/cancers13235927. PMC8657166 34885035

[12] Cesarman E. , Damania B. , Krown S. E. , Martin J. , Bower M. , and Whitby D. , Kaposi Sarcoma, Nature Reviews Disease Primers. (2019) 5, no. 1, 10.1038/s41572-019-0060-9. PMC6685213 30705286

[13] Lee A. J. , Brenner L. , Mourad B. , Monteiro C. , Vega K. J. , and Munoz J. C. , Gastrointestinal Kaposi’s sarcoma: Case Report and Review of the Literature, World Journal of Gastrointestinal Pharmacology and Therapeutics. (2015) 6, no. 3, 89–95, 10.4292/wjgpt.v6.i3.89. PMC4526844 26261737

[14] Bettuzzi T. , Lebbe C. , and Grolleau C. , Modern Approach to Manage Patients With Kaposi Sarcoma, Journal of Medical Virology. (2025) 97, no. 3, 10.1002/jmv.70294. PMC11929155 40119751

[15] Palich R. , Veyri M. , Valantin M. A. et al., Recurrence and Occurrence of Kaposi’s Sarcoma in Patients Living With Human Immunodeficiency Virus (HIV) and on Antiretroviral Therapy, Despite Suppressed HIV Viremia, Clinical Infectious Diseases. (May 23 2020) 70, no. 11, 2435–2438, 10.1093/cid/ciz762. 31626689

